# Development of an Innovative Berberine Food-Grade Formulation with an Ameliorated Absorption: In Vitro Evidence Confirmed by Healthy Human Volunteers Pharmacokinetic Study

**DOI:** 10.1155/2021/7563889

**Published:** 2021-11-27

**Authors:** Giovanna Petrangolini, Fabrizio Corti, Massimo Ronchi, Lolita Arnoldi, Pietro Allegrini, Antonella Riva

**Affiliations:** Research and Development Department, Indena SpA, 20139 Milan, Italy

## Abstract

**Objective:**

To evaluate in vitro solubility, bioaccessibility, and cytotoxic profile, together with a pharmacokinetic profile by oral administration to healthy volunteers of a novel food-grade berberine formulation (BBR-PP, i.e., berberine Phytosome®).

**Results:**

An in vitro increase of solubility in simulated gastric and intestinal fluids and an improved bioaccessibility at intestinal level along with a lower cytotoxicity with respect to berberine were observed with BBR-PP. The pharmacokinetic profile of the oral administration to healthy volunteers confirmed that berberine Phytosome® significantly ameliorated berberine absorption, in comparison to unformulated berberine, without any observed side effects. The berberine plasma concentrations observed with both doses of BBR-PP were significantly higher than those seen after unformulated berberine administration, starting from 45 min (free berberine) and 30 min (total berberine). Furthermore, BBR-PP improved berberine bioavailability (AUC) was significantly higher, around 10 times on molar basis and with observed dose linearity, compared to the unformulated berberine.

**Conclusion:**

These findings open new perspectives on the use of this healthy berberine formulation in metabolic discomforts.

## 1. Introduction

Berberine is a plant-derived quaternary benzylisoquinoline alkaloid with ancient use in the traditional medicine of different countries, especially in China. This compound is found in the root, rhizome, and stem bark of many important plant species such as *Berberis* spp., *Coptis* spp., and *Hydrastis* spp. [1].

Berberine has a long history of use in Ayurveda and Chinese medicine as an antimicrobial, antiprotozoal, antidiarrheal, and antitrachoma agent. A great number of important biological activities were discovered in the last several years by different groups, so the interest in berberine health application as a food supplement and diet component has grown greatly [2]. Berberine has been reported to exhibit antihypertensive, antiarrhythmic, antihyperglycemic, anticancer, antidepressant, anxiolytic, neuroprotective, anti-inflammatory, analgesic, hypolipidemic, nephron and hepatoprotective properties, together with beneficial effects on glycolipid metabolic abnormalities and on regulation of intestinal flora [3–6].

However, the low solubility and the low absorption of berberine itself represent a limiting factor to its activity, so the majority of efforts were addressed to improve those liabilities in order to reduce the high dosages that result in gastrointestinal adverse events [7, 8]. A low intestinal absorption, due to the P glycoprotein (Pgp) efflux, a presystemic degradation by microbiota, and a high hepatic metabolism and renal excretion [9] contribute to the very low berberine bioavailability. A new custom delivery system of berberine based on the Phytosome® technology has been developed and is described in the present paper. Phytosomes are solid dispersions of natural substances and lecithin and represent a food-grade approach of promoting the solubility and capability of poorly bioavailable natural active ingredients to cross biological barriers, leading to a significant improvement in the oral absorption and a consequent amelioration of their biological activity [10, 11]. For each botanical ingredient, there is thorough developmental research in order to achieve the best performing formulation. For these reasons, berberine Phytosome® (BBR-PP) also contains, together with lecithin, pea proteins and a standardized grape seed extract, possibly counteracting Pgp inhibition [12]. The aim of the present research was the evaluation of potential solubility/absorption amelioration of the food-grade berberine formulation by in vitro tests. A pharmacokinetic study was also performed to evaluate the bioavailability of berberine Phytosome® in healthy volunteers in comparison with stand-alone berberine.

## 2. Methods

### 2.1. Formulation

Berberine Phytosome® (BBR-PP, berberine phospholipids/PRO, Patent Application WO2019/150225) is a solid dispersion containing berberine extract in a rational combination with sunflower lecithin, pea protein (NUTRALYS® S85F, supplied by Roquette Freres, Lestrem, France), and grape seed extract. Berberine extract is obtained by aqueous extraction from the roots of *Berberis aristata*. Procedures involved grinding the roots, extraction in an aqueous medium, precipitation, and drying. The *Vitis vinifera* grape seed extract is a proprietary oligomeric proanthocyanidin (OPC) standardized extract (Enovita®) made exclusively with grape seeds from white wine production. Using only water as extraction solvent, grape seed extract is standardized to provide 95.0% of OPCs by spectrophotometry and a relatively low amount of flavane monomers (5.0–15.0% catechin and epicatechin, by HPLC). Finally, food-grade hydroxypropylcellulose (Klucel) and amorphous silica (SYLOID) have been also added to improve the physical and technological properties of the Phytosome® and to facilitate its incorporation in different dosage forms. BBR-PP is standardized to contain 28–34% of berberine (by HPLC).

BBR-PP is obtained by the solvent evaporation method which involves the cosolubilisation and codispersion of berberine extract and of the other components, with the exception of silicon dioxide, in an organic solvent. The organic solvent is then removed under reduced pressure and silicon dioxide is added to the dry powder to improve the flowability during the final calibration step to obtain a suitable granulometry.

### 2.2. Solubility Studies

The solubility of berberine (unformulated and formulated as BBR-PP), in comparison with berberine chloride, was evaluated under the same saturation conditions in the following simulated gastrointestinal media: fasted-state simulated gastric fluid FaSSGF at pH 1.6, fasted-state simulated intestinal fluid FaSSIF at pH 6.5, and fed-state simulated intestinal fluid FeSSIF at pH 5.0. Biological media were prepared according to manufacturer's procedures (Biorelevant.com, London, UK) and the solubility tests were conducted as previously described [10, 11]. Briefly, samples were incubated at the same final amount of berberine (corresponding to 20 mg of berberine chloride) in 10 mL of FaSSGF, FaSSIF, or FeSSIF at room temperature under magnetic stirring for 2 hours. After that period, samples were filtered by 0.2 *μ*m polytetrafluoroethylene (PTFE) syringe to obtain a clear solution and then injected in UPLC (Ultra High Performance Liquid Chromatography), equipped by a Waters ACQUITY H-Class System with PDA Detector. The stationary phase was an ACQUITY CSH C18 column (100 mm × 2.l mm), with particle size of 1.7 *μ*m. Chromatographic analysis was made at 0.4 mL/min flow rate by linear gradient (mobile phase: solvent A: 0.5% phosphoric acid (w/v), solvent B: acetonitrile), according to the following scheme: initial conditions A 95%; at 6 min A 50%; at 6.5 min A 0%; and at 7.5 min A 0%. Column temperature was set at 40°C; autosampler temperature was set at 20°C and detection was performed at 348 nm.

### 2.3. In Vitro Intestinal Studies

Intestinal studies were performed (ECSIN-ECAMRICERT S.r.l., Rovigo, Italy) to evaluate intestinal bioavailability of both BBR-PP formulation and unformulated berberine extract. Berberine bioaccessibility was evaluated as previously described [13], in agreement with EFSA guidelines [14]. Briefly, one dose of each formulation containing the same amount of berberine was subjected to digestion for 3 hours to mimic the digestive physiological process; samples were then centrifuged [15], and the supernatant was analysed by HPLC (see Section 2.4.1). A bioaccessible fraction of berberine released from both formulations during in vitro digestion was expressed as percentage of measured berberine at the end of the digestive process.

The viability of the intestinal cells after treatment with berberine digested samples was evaluated on human colon adenocarcinoma cell line Caco-2 (ATCC, HTB-37TM), cultured on Transwell inserts as already described in detail [13, 16]. Serial dilutions in digestive fluids of the digested formulation were then added to the apical side of the insert, while Hanks' Balanced Salt Solution (HBSS) was added to the basolateral side. After 3 hours of incubation, viability was assessed by MTS colorimetric assay (Promega Italia S.r.l.), based on MTS (3-(4,5-dimetiltiazol-2-il)-5-(3-carboxymethoxyphenyl)-2-(4-solfofenil)-2H-tetrazolium) reduction by vital cells to generate the coloured product formazan, quantified at 490 nm absorbance. As negative control, digestive fluids without berberine were utilized. The percent viability was calculated, and statistical analysis was performed by Student's *t*-test.

### 2.4. Pharmacokinetic Study in Healthy Volunteers

A randomized, double-blind, and crossover pharmacokinetic study of berberine chloride alone (500 mg) in comparison of two doses of BBR-PP administered to healthy volunteers under fasting conditions was conducted at the Araba University Hospital (Vitoria-Gasteiz, Spain).

Twelve healthy volunteers (after screening of 24) of both sexes with an age range of 18–50 inclusive, with BMI 18.5–25, were recruited. No withdrawal occurred in the trial.

No medication or herbal products were allowed within 15 days prior to the date of the study. Only authorized medication in exceptional cases was contemplated, as long as its pharmacokinetic profile did not interfere with that of the treatment being tests, and subsequently evaluated and reported. Alcohol-containing and stimulating beverages/foods (such as coffee, tea, chocolates, and soft drinks) were abstained from, at least from selection visit until the end of study; no water was consumed up to one hour after administration of study treatment, and no food was allowed up to four hours after administration.

Inclusion criteria were no evidence of significant organic or psychiatric disease, based on history, physical examination, and additional tests, and laboratory tests (blood count, biochemistry and elementary and urine sediment) within normal range, according to normal reference values of the Laboratory of Biochemistry, University Araba Hospital, Txagorritxu headquarters in Vitoria-Gasteiz. A negative serology for HBV, HCV, and HIV and vital signs (BP, HR, RR, and temperature) and ECG within normal limits were required.

The study was carried out in accordance with the Declaration of Helsinki (1964) guidelines and its amendments, and the general principles of ICH Harmonised Tripartite Guidelines for Good Clinical Practice (ICH Topic E6, CPMP/ICH/135/95). At the beginning of the study, written informed consent, reviewed by the Ethics Committee, was obtained from each subject, according to ICH-GCP, the ethical principles that have their origin in the Declaration of Helsinki, and the regulatory and legal requirements in Spain. The Spanish Research Ethics Committee of Araba University Hospital (Vitoria-Gasteiz) approved the study on May 23, 2019 (protocol code IDN_BERB01/19).

#### 2.4.1. Study Design and Treatments

Treatments administered were as follows:

- A: Berberine chloride 500 mg, one film-coated tablet (equivalent to 452 mg berberine)
- B: BBR-PP 550 mg (equivalent to 188 mg berberine), one film-coated tablet
- C: BBR-PP 550 mg (equivalent to 188 mg berberine), two film-coated tablets

Berberine 500 mg/dose was formulated as film-coated tablets containing the following ingredients: dicalcium phosphate dihydrate, microcrystalline cellulose, magnesium carbonate, sodium croscarmellose, silicon dioxide, talc, magnesium stearate, and hydroxypropylmethylcellulose-based film-coating.

BBR-PP 550 mg/dose was formulated as film-coated tablets containing the following food-grade ingredients: dicalcium phosphate dihydrate, microcrystalline cellulose, sodium croscarmellose, silicon dioxide, talc, magnesium stearate, and hydroxypropylmethylcellulose-based film-coating.

Film-coated tablets of both BBR-PP and berberine were characterized for appearance, average mass, uniformity of mass, HPLC content of berberine, disintegration time, and microbiological quality.

Each volunteer received treatments A, B, and C, with a 7-day washout interval between treatments, and the sequence or order of administration was randomized. This process was carried out using a list of random numbers generated by the MAS program version 2.1 (sampling and random assignment) included in the software package Design C4 Study Pack developed by the Department of Biometrics/Medical of GlaxoSmithKline (Brentford Middlesex, United Kingdom).

Oral administration was performed in fasted-state condition early on each experimental day, with 200 mL of water. Blood samples (5 mL each), obtained by inserting an indwelling cannula for blood sample collection in a forearm vein, were collected at 12 time points from each subject at each study period as follows: one predose sample and 11 after dosing at the following time points: +15 min, +30 min, +45 min, +1 h, +2 h, +3 h, +4 h, +6 h, +8 h, +12 h, and +24 h after administration of the treatment. Blood was transferred into prelabelled EDTA tubes and centrifuged at 4°C for 10 minutes at 2000 g within one hour from collection. Baseline plasma samples and those obtained after administration of treatment were stored at −20°C. At the end of the first trial day (after the +12 h blood extraction), all samples from the volunteers were stored at −80°C until analysis. The qualification was carried out at Kymos Pharma Services S.L. (Barcelona, Spain), according to the guidelines OCDER Industry [17] and guideline on bioanalytical method validation [18], studying the following validation parameters: (a) selectivity, (b) calibration curve (linearity), (c) lower limit of quantification (LLOQ), (d) intra-assay accuracy and precision, (e) carryover, and (f) system suitability test. Based on 200 *μ*L plasma volume (K2-EDTA as anticoagulant), the determination of total and free berberine in human samples was carried out by HPLC-MS/MS, followed by a liquid-liquid extraction with ethyl acetate. Samples for total berberine were previously subjected to enzymatic hydrolysis with sulfatase from *Helix pomatia* and *β*-Glucuronidase from bovine liver, while samples for free berberine were not subjected to hydrolysis. Compound berberine-d6 was used as internal standard. The method was checked for linearity, precision, and accuracy, within the range from 10 to 5000 pg/mL of total berberine and within the range from 5 to 500 pg/mL of free berberine. Peak area ratios were fitted according to a linear model (1/*χ*2 weighing, with correlation coefficients higher than 0.99 and the intercepts close to zero); blanks (blank human plasma), zero sample (blank spiked with internal standard), and quality control samples at four different concentrations (nominal concentrations of 30, 250, 2500, and 4000 pg/mL) were included. Sample analysis was performed by HPLC (Agilent 1290, Infinity) coupled to Mass Spectrometer Triple Quadrupole 6500 (SCIEX, US), equipped with a TurbolonSpray Ion source. For HPLC, a 3 *μ*m Atlantis dC18 column (2.1 × 100 mm, Waters) was eluted at constant flow of 0.4 mL/min and temperature of 40°C, with a gradient of 10 mM ammonium acetate (0.1%formic acid) as solvent A and acetonitrile (0.1% formic acid) as solvent B, according to the following scheme: initial conditions A 85% and B 15%; at 4 min A 10% and B 90%; at 5 min A 10% and B 90%; at 5.1 min A 85% and B 15%; and at 7 min A 85% and B 15%. Quantification was carried out using positive multiple reaction monitoring (MRM) at *m*/*z* 336.1 ⟶ 320.1 (berberine) and at *m*/*z* 342.1 ⟶ 322.1 (berberine-d6) transitions.

#### 2.4.2. Safety and Tolerability

Clinical safety (evaluation of vital signs and systemic adverse effects) and biological safety (evaluation of subjects' blood count and blood chemistry) were tested.

#### 2.4.3. Data Analysis

A noncompartmental pharmacokinetic analysis of total and free berberine was carried out using individual concentrations, theoretical doses, and actual sampling times by means of the validated software WinNonlin program. The following pharmacokinetic parameters were estimated: AUC_last_ (area under the plasma concentration versus time curve from time zero to the time of the last quantifiable concentration), *C*_max_ (maximum plasma concentration), *T*_max_ (time to achieve *C*_max_), and mean residence time (MRT). Statistical analysis was performed by one-way and two-way ANOVA for repeated measures, followed by Tukey's test.

## 3. Results

### 3.1. Solubility Studies

Solubility in vitro studies were performed in fasted-state simulated gastric fluid (FaSSGF) at pH 1.6, FaSSIF (fasted-state simulated intestinal fluid) at pH 6.5, and FeSSIF (fed-state simulated intestinal fluid) at pH 5.0 on BBR-PP, in comparison with berberine chloride and the unformulated berberine, that is, the berberine extract. A 3-4-fold increase in solubility was observed for BBR-PP versus berberine extract in the three fluids, as reported in Table 1.

Also, in the case of berberine chloride, an increase in solubility in FASSGF of about 6-fold was observed when pea proteins and *Vitis vinifera* seed extract were included in BBR-PP (data not shown). After those encouraging results, it was decided to address further studies on the most soluble formulation, BBR-PP.

### 3.2. In Vitro Intestinal Studies

**Figure 1:**
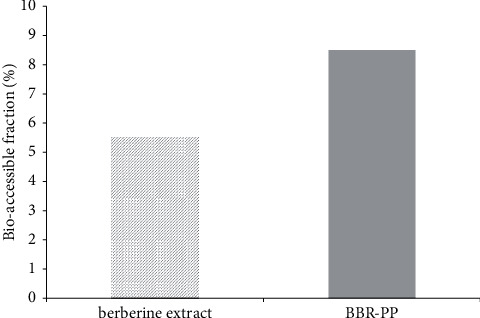
Bioaccessibility of berberine extract and BBR-PP in Caco-2 intestinal cell lines.

The bioaccessibility of BBR-PP, in comparison with berberine extract, was determined after exposing one dose to the in vitro digestive process to mimic the in vivo physiological conditions. The term bioaccessibility refers to bioactive compounds and nutrients and represents the amount of an ingested nutrient that should be released from a matrix becoming available to be absorbed after undergoing the digestion process, as previously described [19]. Results obtained suggest that almost all of berberine remains at the end of the digestion for both products (Table 2). In terms of bioaccessibility, that is, the soluble fraction released from the matrix and available for the absorption, BBR-PP showed an improvement with respect to the unformulated berberine extract (Table 2 and Figure 1).

The bioaccessibility fraction was expressed as percentage of measured berberine at the end of the digestive process.

**Figure 2:**
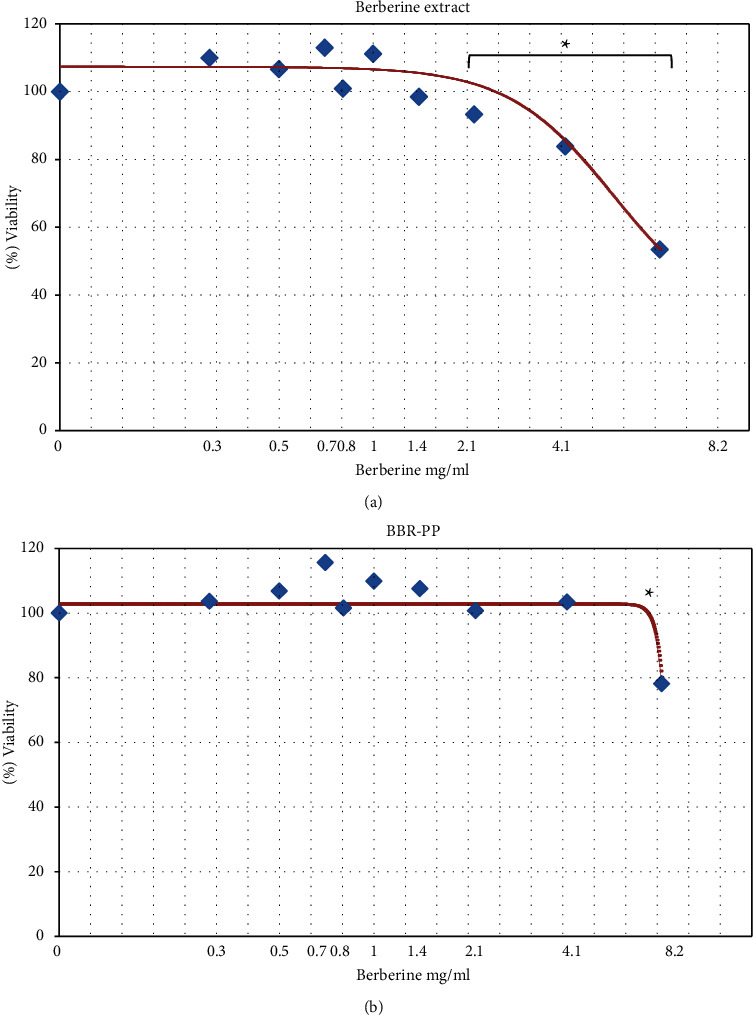
Effect of berberine extract (a) and BBR-PP (b) on Caco-2 intestinal viability.

The cytotoxic potential of both berberine extract and BBR-PP was evaluated after incubation for 3 h in Transwell-plated Caco-2 cells, the well-known model widely utilized in literature [20]. As shown in Figure 2, BBR-PP is not toxic at all in the tested dilutions up to 4.5 mg/mL berberine final concentration, even if a 22% inhibition of viability was observed at the highest concentration tested (8.2 mg/mL). However, berberine extract significantly inhibited viability at only 2.1 mg/mL and reached about 50% inhibition of viability at 8.2 mg/mL final concentration.

Viability was evaluated by MTS assay. Data are expressed as means of experiments performed in triplicate, and each experiment was repeated three times; ^*∗*^*p* < 0.05.

In order to confirm the safety of BBR-PP before conducting the human study, a Good Laboratory Practice (GLP) limit acute toxicity test (data not shown) was performed according to OECD guideline and EU and national regulatory law on animal welfare [21], indicating that BBR-PP does not induce toxic effects following oral administration of a single 2000 mg/kg dose. The lack of mortality and no adverse clinical signs in rats demonstrated the acute toxicity expected (ATE) to be greater than 2000 mg/kg body weight.

### 3.3. Pharmacokinetic Study in Healthy Volunteers

A randomized and crossover pharmacokinetic study of berberine chloride (the reference form of berberine used in dietary supplements) as a 500 mg film-coated tablet (equivalent to 452 mg berberine) in comparison with two doses of BBR-PP (550 mg one and two film-coated tablets, equivalent to 188 mg berberine each) was performed in healthy volunteers under fasting conditions.

**Figure 3:**
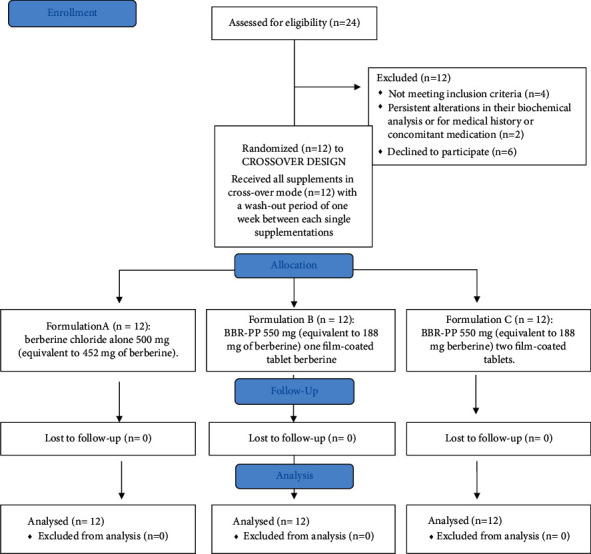
Study flow diagram.

Tablets were prepared using a direct compression process, and each tablet contained different excipients: as diluents: calcium phosphate, microcrystalline cellulose, magnesium stearate, or carbonate; as disintegrants: sodium croscarmellose; and as lubricant, flowing agents: talc and silica. Both tablet formulations have been coated with a ready-to-use hydroxypropylmethylcellulose-based film coating, in order to mask the two different formulations and allow the blindness of the study between the different supplementations. Both tablet formulations were successfully tested for berberine content, average weight, uniformity of mass, disintegration time (resulting ≤30 minutes), heavy metals, and microbiological quality. A total of 24 subjects were examined during recruitment; 12 subjects (9 females and 3 males) initiated the study and all of them completed it, as reported in the following study flow diagram (Figure 3).

All the collected plasma samples from the study were analysed in five valid chromatographic batches for berberine determination, and each one included a set of calibration standards (concentration range from 10 to 5000 pg/mL). The analytical method was selective in human plasma, linear, precise, and accurate within the range from 10 to 5000 pg/mL of total berberine with the lower limit of quantitation (LLOQ) of 10 pg/mL and within the range from 5 to 500 pg/mL for free berberine with the LLOQ of 5 pg/mL. The mean accuracies of the back calculated concentrations, expressed as relative error, ranged between -5.11% and 4.30% and the precision was below or equal to 5.98%. Calibration curve and the original chromatograms are available in Supplementary Figures 1 and 2. The mean accuracies, expressed as relative error, of the quality control samples ranged between 1.17% and 7.12% and the precision was below or equal to 5.72%.

**Figure 4:**
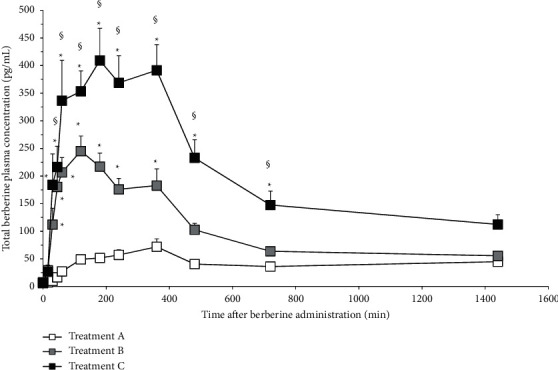
Pharmacokinetic profile of total berberine in clinical study. Statistical analysis was performed by two-way ANOVA, followed by Tukey's test, ^*∗*^*p* < 0.05 versus A; ^§^*p* < 0.05 versus B. Symbols represent mean values (±standard deviation). Formulations: A: berberine chloride alone 500 mg (equivalent to 452 mg of berberine); B: BBR-PP 550 mg (equivalent to 188 mg of berberine) one film-coated tablet; C: BBR-PP 550 mg (equivalent to 188 mg berberine) two film-coated tablets. Twelve healthy volunteers of both sexes with a mean age of 29 ± 7.72 and a mean BMI of 23.09 ± 1.27 were enrolled.

**Figure 5:**
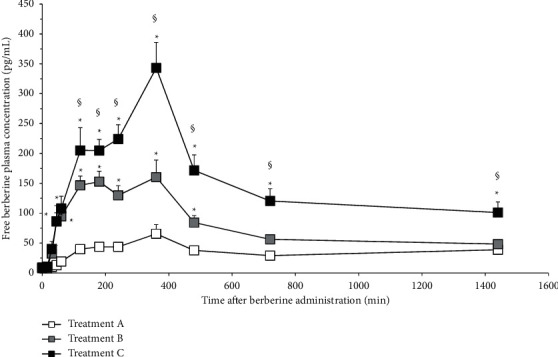
Pharmacokinetic profile of free berberine in clinical study. Statistical analysis was performed by two-way ANOVA, followed by Tukey's test, ^*∗*^*p* < 0.05 versus A; ^§^*p* < 0.05 versus B. Symbols represent mean values (±standard deviation). Formulations: A: berberine chloride alone 500 mg (equivalent to 452 mg of berberine); B: BBR-PP 550 mg (equivalent to 188 mg of berberine) one film-coated tablet; C: BBR-PP 550 mg (equivalent to 188 mg berberine) two film-coated tablets. Twelve healthy volunteers of both sexes with a mean age of 29 ± 7.72 and a mean BMI of 23.09 ± 1.27 were enrolled.

The pharmacokinetic profile of total and free berberine after administration of BBR-PP and berberine chloride to healthy volunteers is shown in Figures 4 and 5, respectively.

Total berberine concentration is considered as the sum of free and conjugated fractions. The berberine plasma concentrations observed with both doses of BBR-PP were significantly higher than those seen after unformulated berberine administration, starting from 45 min (free berberine) and 30 min (total berberine). Looking at the BBR-PP kinetic curves after one (B) or two (C) daily supplementations with BBR-PP, it is possible to observe that berberine plasma concentrations are high and remain stable for several hours after oral administration. After 6 hours, almost all the berberine is under the free form, suggesting that extensive metabolism reached a peak 6 hours after administration. So the new delivery food-grade formulation allows interesting plasmatic levels of the free form that can be available to reach target tissues. 24 hours after administration of BBR-PP, berberine is still detectable at plasma level.

In terms of calculated pharmacokinetic parameters (Table 3), the AUC_last_ values obtained from the BBR-PP treatment, one tablet and two tablets, are significantly higher than those obtained from the unformulated berberine.

The AUC_last_ value of BBR-PP tablet is about 4 times (for total berberine) and 2.4 times (for free berberine) higher than values from berberine chloride. Considering that the ratio of effective berberine content in formulated versus unformulated tablet is about 2.4, the real improvement in AUC_last_ BBR-PP (considering total berberine) is of 9.76 and 6 times (considering free berberine) with respect to berberine chloride. A dose linearity was observed, based on the two doses administered, as seen by the AUC_last_ values displayed. When *C*_max_ values are considered, a profile similar to that of AUC_last_ was observed, with a maximal (7-fold) increase in *C*_max_ by two tablets of BBR-PP supplementation as far as total berberine is concerned. *T*_max_ values were not significantly different among groups considering total berberine, while a significant reduction (free berberine data) can be observed for BBR-PP one tablet versus unformulated berberine and BBR-PP two tablets. MRT can be calculated only for free berberine and both one- and two-tablet supplementations are statistically higher values compared to the unformulated berberine. BBR-PP formulation allows persistent plasma levels of berberine, better than unformulated berberine.

No clinical and biological issues during the study in all the groups were detected, indicating that the treatment was safe and well tolerated.

## 4. Discussion

With the present study, and for the first time, a new food-grade bioavailable custom delivery system of berberine formulated in Phytosome® was developed (BBR-PP). This new ingredient showed an improved solubility with respect to unformulated berberine confirmed as a significant amelioration of berberine bioavailability in healthy volunteers, correlated to a good tolerability, good compliance, and, of note, a better safety profile.

A growing interest in berberine and its properties has emerged in recent years, although its use in traditional Chinese and Indian medicine has long been well known [1, 22]. The potential usefulness of berberine in a variety of human diseases has been described by many research papers: from type II diabetes [23, 24] due to berberine glucose-lowering properties to metabolic syndrome and hyperlipemia [25, 26] and bacterial diseases due to the antimicrobial properties shown versus different microorganisms [27, 28].

In order to overcome the drawbacks of the well-known low solubility and low oral absorption of berberine [9], formulative efforts by using different techniques were developed. Very recently, nanostructured lipid carriers (NLCs) were described as more effective than berberine in DSS-induced colitis model in mice [29]; microspheres containing absorption enhancer (sodium N-[8-(2-hydroxybenzoyl) amino]caprylate (SNAC) improved berberine oral absorption in rats [16]. By using a mixed micelle formulation with pluronic acid P85 and tween 80, Kwon et al [30] obtained a more bioavailable berberine.

The application of the food-grade Phytosome® delivery system to improve bioavailability of berberine, described in the present study, was conceived on the basis of previous successful examples with other natural substances like quercetin and CoQ10 [10, 11]. Each new formulation in Phytosome® has to be adapted to the properties of the specific natural compound under consideration. Raw extract has been used in order to better match the natural composition of *Berberis aristata* roots. In the case of BBR-PP, the specific formulation design required the addition to the lecithin matrix of two more ingredients, that is, pea proteins and *Vitis vinifera* seed extract, to give rise to a more soluble and more bioavailable product.

In vitro studies in the gastrointestinal fluid FaSSGF showed a substantial improvement compared to the unformulated berberine in terms of solubility up to sixfold when berberine is combined with pea proteins and *Vitis vinifera* seed extract. Positive results for BBR-PP were also obtained in intestinal FaSSIF and FeSSIF fluids. When tested on the classical intestinal cell model Caco-2, BBR-PP showed better bioaccessibility than berberine extract (8.5% versus 5%). Furthermore, the minor toxicological impact on intestinal mucosa seen with BBR-PP is particularly appealing, due to the known long-term side effects described after chronic administration of berberine. Therefore, a formulation less toxic than berberine chloride should be more tolerated especially for long-term administration, where gastrointestinal adverse events were observed [31]. Moreover, the presence of *Vitis vinifera* seed extract in BBR-PP should support the final supplement thanks to its antioxidant and protective properties [32].

Most importantly, when BBR-PP was orally administered to healthy volunteers, the advantages in terms of solubility and bioaccessibility seen in vitro are reflected in a highly significant increase in plasma berberine concentrations versus the unformulated berberine. A 4-fold (one tablet) and 6-fold (two tablets) increase in AUC (calculated on total berberine) with respect to berberine chloride treated group was observed after BBR-PP administration. That increase is up to tenfold by considering the effective berberine content in tablets (452 mg for berberine chloride and 188 mg for BBR-PP). Furthermore, a dose-dependent increase in AUC_last_ was suggested considering BBR-PP one-tablet and two-tablet groups. At 24 hours after treatment with BBR-PP, berberine plasma levels are quite detectable and allow the assumption that, with repeated administration, it is possible to achieve the steady state.

Moreover, the differences observed between the two formulations are evident when both total berberine and free berberine are analysed. In fact, berberine exerts its action both systemically and at the liver level; it can therefore be argued that the free plasma fraction is representative of the systemic action (such as hormones and insulin resistance), while the conjugated fraction (where total represents the sum of free and conjugated) may exert its relevant metabolic activity in the liver (AMPK, PCSK9, and lipidic metabolism). The two activities are synergic and for this reason both free and total berberine levels are relevant for the human benefit [33]. Differences in the ratio of AUC total/free observed in BBR-PP and unformulated berberine might be attributed to a different absorption due to the different formulations. A possible limitation for the present pharmacokinetic study would be the number of participants (12), even if randomization, double-blindness, and crossover were included in the study design.

No major side effects were observed during the human study, indicating that the new custom formulation was well tolerated with a good safety profile.

Further studies are now ongoing to support the health benefits of this new food-grade delivery system in order to explore the great potential of BBR-PP in dysmetabolic syndromes.

## 5. Conclusions

A new food-grade custom formulation of berberine (berberine Phytosome®) was proposed as more soluble and more absorbed than unformulated berberine. Comparative data after oral administration in healthy volunteers confirmed the in vitro studies. The pharmacokinetic profile of BBR-PP was highly superior to the unformulated one in terms of berberine bioabsorption with a proved safety, suggesting good prospective of its use in dysmetabolic and gastrointestinal unhealthy conditions.

## Figures and Tables

**Table 1:** Solubility studies in gastric simulated fluids.

Material	FaSSGF	FeSSIF	FaSSIF
	Berberine (mg/mL)		
BBR-PP	0.603	0.329	0.365
Berberine extract	0.188	0.129	0.149

Solubility studies were performed in FaSSGF (fasted-state simulated gastric fluid) at pH 1.6; FaSSIF (fasted-state simulated intestinal fluid) at pH 6.5, or FeSSIF (fed-state simulated intestinal fluid) at pH 5.0.

**Table 2:** In vitro digestion.

Material	% after digestion^*∗*^	% bioaccessible fraction^*∗∗*^
Berberine extract	94.5 ± 0.6	5.5
BBR-PP	91.5 ± 0.7	8.5

^*∗*^Values are expressed as means ± standard deviations. ^*∗∗*^Values are expressed as percentage of measured berberine at the end of the digestive process.

**Table 3:** Pharmacokinetic parameters.

Parameters	A	B	C
*Free berberine*			
AUC_last_ (pg/mL^∗^h)	1057 ± 117	2544 ± 332^*∗*^	4146 ± 431^∗§^
*C*_max_ (pg/mL)	69.95 ± 14.54	199.87 ± 26.97^*∗*^	375.57 ± 41.56^∗^^§^
*T*_max_ (h)	4.55 ± 0.29	3.15 ± 0.30^*#∗*^	4.50 ± 0.30
MRT (h)	12.13 ± 0.19	9.16 ± 0.17^*∗*^	9.53 ± 0.26^*∗*^

*Total berberine*			
AUC_last_ (pg/mL^∗^h)	1217 ± 129	4952 ± 647^*∗*^	8212 ± 893^∗^^§^
*C*_max_ (pg/mL)	76.70 ± 14.04	316.88 ± 26.60^*∗*^	572.14 ± 64.26^∗^^§^
*T*_max_ (h)	3.59 ± 0.34	2.13 ± 0.33	3.10 ± 0.34
MRT(h)	NC	NC	NC

Results are expressed as mean ± S.E.M.; NC: not calculable. Statistical analysis was performed by one-way ANOVA, followed by Tukey's test. ^*∗*^*p* < 0.05 versus A, ^§^*p* < 0.05 versus B, and ^#^*p* < 0.05 versus C. Formulations: A: berberine chloride alone 500 mg (equivalent to 452 mg of berberine); B: BBR-PP 550 mg (equivalent to 188 mg of berberine) one film-coated tablet; C: BBR-PP 550 mg (equivalent to 188 mg berberine) two film-coated tablets. Twelve healthy volunteers of both sexes with a mean age of 29 ± 7.72 and a mean BMI of 23.09 ± 1.27 were enrolled.

## Data Availability

The data used to support the findings of this study are available from the corresponding author upon request.

## Supplementary Materials

Supplementary Materials Supplementary Figures 1 and 2 describe calibration curve and the original chromatograms obtained by analysis of clinical study's samples.
