# Supplementary figures and images for: Development of an Innovative Berberine Food-Grade Formulation with an Ameliorated Absorption: In Vitro Evidence Confirmed by Healthy Human Volunteers Pharmacokinetic Study
Source: Evid Based Complement Alternat Med. 2021 Nov 27;2021:7563889. doi: 10.1155/2021/7563889 (PMC8665891; doi:10.1155/2021/7563889)

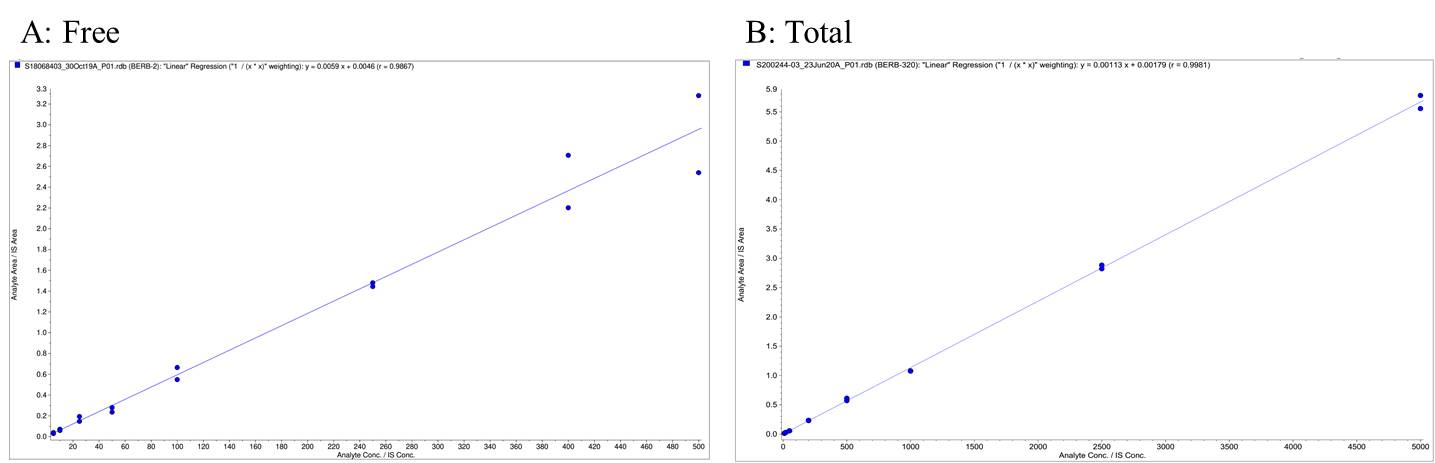

Supplement: Supplementary Materials — Supplementary Figures 1 and 2 describe calibration curve and the original chromatograms obtained by analysis of clinical study's samples. [file 7563889.f1.zip › 7563889.f1/Riva et al_Suppl. Figure 1.jpg]

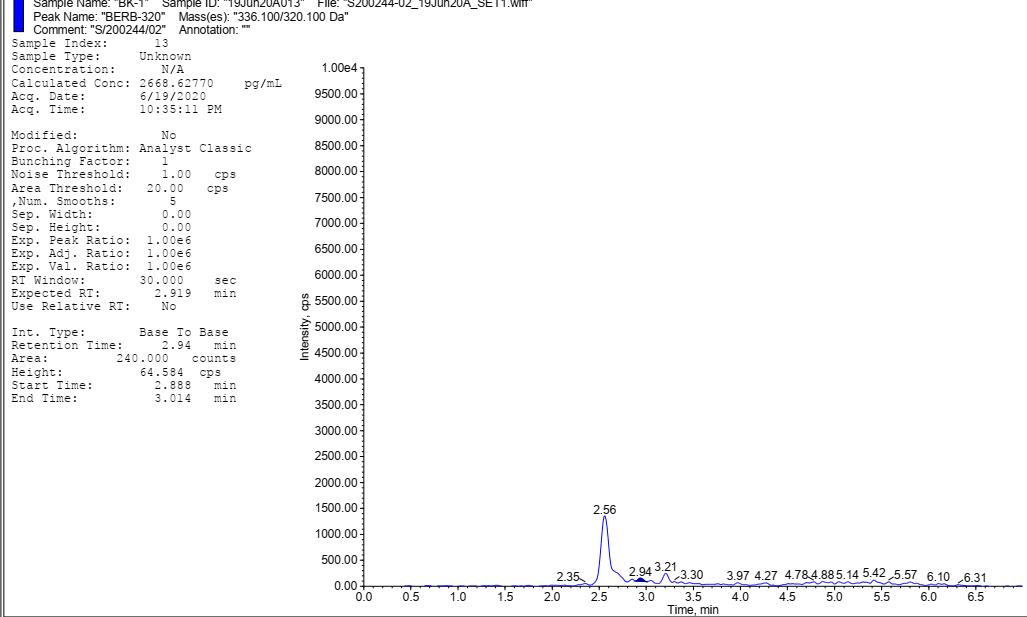

Supplement: Supplementary Materials — Supplementary Figures 1 and 2 describe calibration curve and the original chromatograms obtained by analysis of clinical study's samples. [file 7563889.f1.zip › 7563889.f1/Riva et al_Suppl. Figure 2-A.jpg]

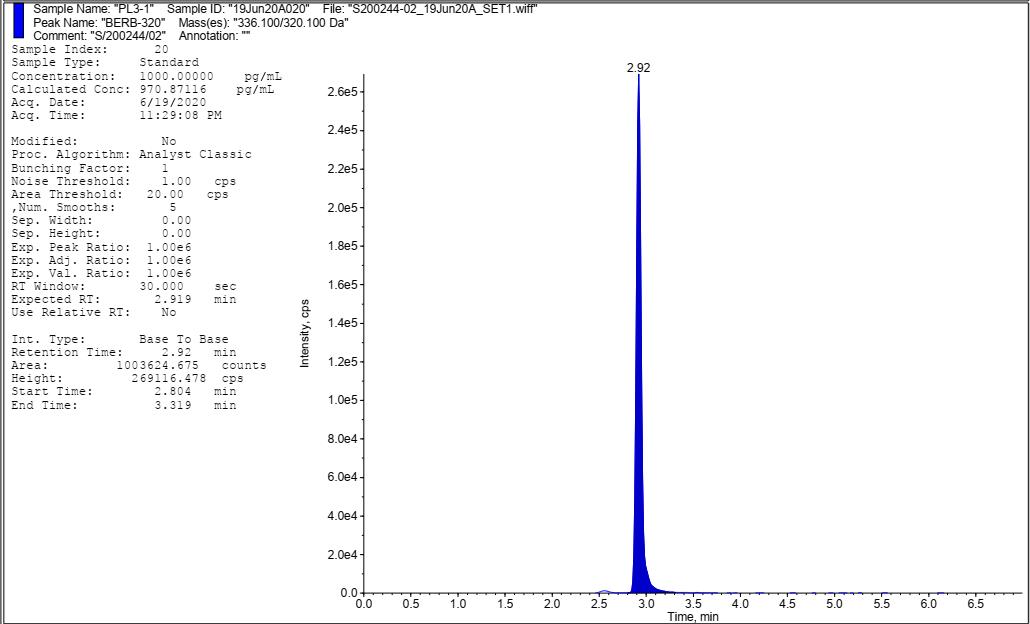

Supplement: Supplementary Materials — Supplementary Figures 1 and 2 describe calibration curve and the original chromatograms obtained by analysis of clinical study's samples. [file 7563889.f1.zip › 7563889.f1/Riva et al_Suppl. Figure 2-B.jpg]

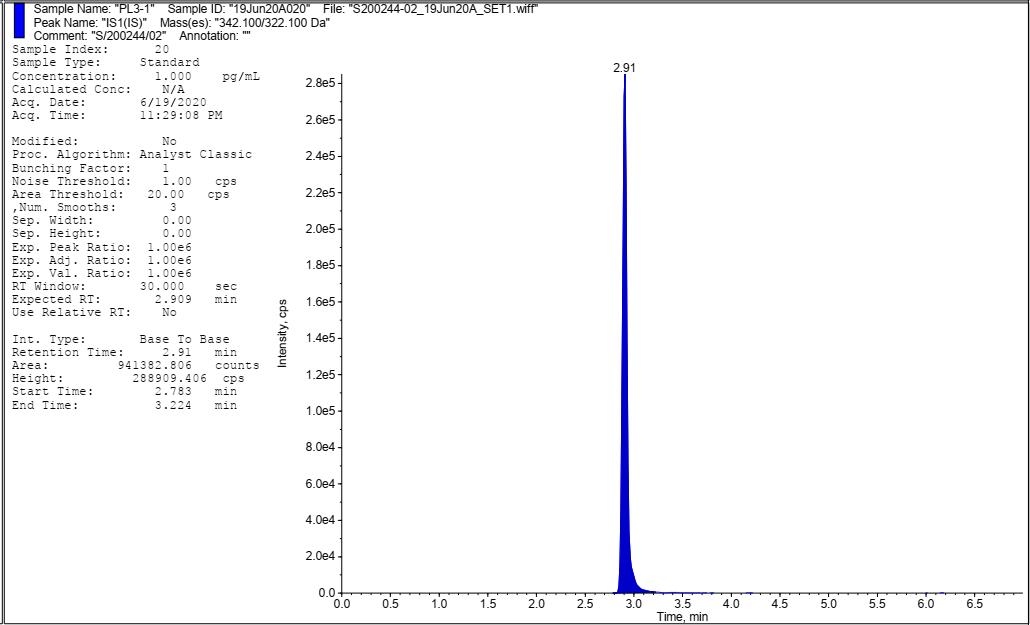

Supplement: Supplementary Materials — Supplementary Figures 1 and 2 describe calibration curve and the original chromatograms obtained by analysis of clinical study's samples. [file 7563889.f1.zip › 7563889.f1/Riva et al_Suppl. Figure 2-C.jpg]
